# Assessment of Enterovirus Antibodies during Early Childhood Using a Multiplex Immunoassay

**DOI:** 10.1128/spectrum.05352-22

**Published:** 2023-05-25

**Authors:** N. V. V. Jouppila, J. Lehtonen, E. Seppälä, L. Puustinen, S. Oikarinen, O. H. Laitinen, M. Knip, H. Hyöty, V. P. Hytönen

**Affiliations:** a Faculty of Medicine and Health Technology, Tampere University, Tampere, Finland; b Research Program for Clinical and Molecular Metabolism, Faculty of Medicine, University of Helsinki, Helsinki, Finland; c Department of Pediatrics, Tampere University Hospital, Tampere, Finland; d Fimlab Laboratories, Tampere, Finland; Keck School of Medicine of the University of Southern California

**Keywords:** enterovirus, children, humoral response, antigen, multiplex assay, diagnostics, serology

## Abstract

Enteroviruses are a group of positive single-stranded viruses that belong to the Picornaviridae family. They regularly infect humans and cause symptoms ranging from the common cold and hand-foot-and-mouth disease to life-threatening conditions, such as dilated cardiomyopathy and poliomyelitis. Enteroviruses have also been associated with chronic immune-mediated diseases, such as type 1 diabetes, celiac disease, and asthma. Studying these disease-pathogen connections is challenging due to the high prevalence of enterovirus infections in the population and the transient appearance of the virus during the acute infection phase, which limit the identification of the causative agent via methods based on the virus genome. Serological assays can detect the antibodies induced by acute and past infections, which is useful when direct virus detection is not possible. We describe in this immuno-epidemiological study how the antibody levels against VP1 proteins from eight different enterovirus types, representing all seven of the human infecting enterovirus species, vary over time. VP1 responses first significantly (*P* < 0.001) decline until 6 months of age, reflecting maternal antibodies, and they then start to increase as the infections accumulate and the immune system develops. All 58 children in this study were selected from the DiabImmnune cohort for having PCR-confirmed enterovirus infections. Additionally, we show that there is great, although not complete, cross-reactivity of VP1 proteins from different enteroviruses and that the response against 3C-pro could reasonably well reflect the recent Enterovirus infection history (ρ = 0.94, *P* = 0.017). The serological analysis of enterovirus antibodies in sera from children paves the way for the development of tools for monitoring the Enterovirus epidemics and associated diseases.

**IMPORTANCE** Enteroviruses cause a wide variety of symptoms ranging from a mild rash and the common cold to paralyzing poliomyelitis. While enteroviruses are among the most common human pathogens, there is a need for new, affordable serological assays with which to study pathogen-disease connections in large cohorts, as enteroviruses have been linked to several chronic illnesses, such as type 1 diabetes mellitus and asthma exacerbations. However, proving causality remains an issue. In this study, we describe the use of an easily customizable multiplexed assay that is based on structural and nonstructural enterovirus proteins to study antibody responses in a cohort of 58 children from birth to 3 years of age. We demonstrate how declining maternal antibody levels can obscure the serological detection of enteroviruses before the age of six months and how antibody responses to nonstructural enterovirus proteins could be interesting targets for serodiagnosis.

## INTRODUCTION

Enteroviruses (EVs) are a group of positive single-stranded viruses that belong to the Picornaviridae family. They regularly infect humans and cause symptoms ranging from the common cold and hand-foot-and-mouth disease to life-threatening conditions, such as dilated cardiomyopathy and poliomyelitis (1–3). Enteroviruses have also been associated with chronic immune-mediated diseases, such as type 1 diabetes (T1D), celiac disease, and asthma. Yearly, the costs of productivity losses due to the common cold are estimated to be about 25 billion dollars, and the estimated total cost of T1D is 14.4 billion dollars in the USA alone (4, 5). By the current definition of the International Committee on the Taxonomy of Viruses (ICTV), there are seven human infecting enterovirus species: Enterovirus A through Enterovirus D and Rhinovirus A through Rhinovirus C.

Enterovirus infections follow a seasonal pattern, the peaks of which typically coincide between early summer and autumn in temperate parts of the world (6), except for Rhinovirus C, which peaks in winter. Children are more susceptible to rhinovirus infections, as they experience on an average 8 to 12 rhinovirus infections each year, whereas adults suffer only 2 to 3 (7, 8).

Enteroviruses may occur as epidemics or as endemics with more stable incidences over time, depending on the virus type. As an example, EV-A71 and EV-68 have caused widespread epidemics roughly every 2 years (EV-A71 in Asia and EV-D68 in Europe and the USA), the most recent of which occurred in multiple countries in 2022 after the COVID-19 lockdowns (9–12). While enterovirus infections and, especially, rhinovirus infections are among the most common virus infections in young children, new types are continuously discovered. Indeed, the discovery of Rhinovirus C viruses is quite recent (13), and Rhinovirus C infections are currently reported to be the causative agents in up to 41% of the acute respiratory infections that require a visit to the pediatric intensive care unit (3).

At present, enterovirus infections are frequently diagnosed using PCR-based methods that target a highly conserved 5′ untranslated region (5’UTR) of the virus genome. However, this method is not able to distinguish different enterovirus types or species from each other. The amplification and sequencing of less conserved regions of the viral genome have been used to identify viral subtypes, but their sensitivity is not optimal for the detection of small amounts of virus. As most enteroviruses cause similar symptoms and because there are no clinically approved antivirals for enteroviruses, the information about exact infecting species is not considered necessary for clinical care, unless there is an epidemic going on. However, the identification of viral subtypes is important in studies addressing the roles of enteroviruses in chronic diseases in epidemiological surveys. Various enteroviruses are known to infect different anatomical sites, primarily intestinal or respiratory mucosa, and, in addition to these primary replication sites, they can infect internal organs, including the heart, central nervous system, or pancreas, which leads to variable sequelae (8, 14).

Serological methods have certain important advantages, compared to direct virus detection. The most important one is that they can detect the infections even when the virus has already disappeared. In many cases, antibodies stay elevated long after the infection, representing a serological scar of past infections. An analysis of neutralizing antibodies can identify the exact enterovirus type that has caused the infection, but the assay is labor-intensive, expensive, and possible for only those enteroviruses which replicate in cell culture. Enzyme-linked immunoassays are faster, but there is considerable cross-reactivity between non-neutralizing antibodies that target different enteroviruses (15–17) and the general population, which has high levels of antibodies (18) due to the high prevalence of different kinds of enteroviruses. However, in our previous studies, we have observed that the antibody responses toward viral envelope protein 1 (VP1) proteins are more cross-reactive in adults than in children (19). We have also shown that the antibody responses toward enterovirus nonstructural protein proteases 2A and 3C are potential markers of acute infection (20).

This study set out to find out how the antibody responses toward enteroviral VP1 proteins and proteases change during the first 3 years of life and how widely different enterovirus types cross-react when structural and nonstructural proteins from different enterovirus species are used as antigens. As expected, we observed the levels of maternally transferred antibodies to steadily decline for approximately 6 months after birth, after which the antibody levels against enteroviral antigens started to increase again, as the children experienced enterovirus infections. We saw that there was cross-reactivity between the VP1 proteins that represented different enterovirus types when looking for trends across samples from multiple children, but, for individual subjects, the antibody responses were more specific. The response against 3C appeared to be a suitable measure by which to evaluate the overall enterovirus infection load of the patient. As an inactivated poliovirus vaccine is used in many counties in young children, it generates confounding background signals with structural protein antigens. Since the vaccine does not include viral proteases or other nonstructural proteins, antibodies against proteases are potential candidates for the analysis of the true infection load, as they are not present in inactivated poliovirus or enterovirus 71 vaccines. Similar approaches that rely on nonstructural proteins are commonly used in the cattle industry for foot and mouth disease (FMD) (21).

## RESULTS

We took a top-down approach to understand the antibody responses toward enteroviruses. First, we analyzed the responses over all of the subjects (Table 1) and viral species to understand how the responses developed in children. Based on this information, we moved on to analyze data at the enterovirus group (species) level. Lastly, we compared the trends observed on the infection group level to those seen in individual patients.

**TABLE 1:** Description of the human samples and their grouping, according to infections observed in enterovirus RT-PCR and sequencing analyses

Infection group (EV species)	Species/types detected by sequencing (*n*)	Total number of PCR positive subjects	Representative VP1 antigen used in the current antibody analyses
Enterovirus A	CVA16 (3), CVA2 (1), CVA4 (14), CVA6 (3), CVA8 (1), EV-A71 (1)	23	Coxsackievirus A4 (CVA4)
Enterovirus B	CVA9 (2), CVB1 (1), CVB3 (2), CVB5 (1), E30 (1)	7	Coxsackievirus B1 (CVB1)
Enterovirus C	Polio1 (1)	1	Poliovirus 1 (PV1)
Enterovirus D	EV-D68 (13)	13	Enterovirus D68 (EV-D68)
Rhinovirus A	RV-A (3), RV-A103 (1), RV-A45 (1), RV-A51 (1), RV-A53 (1), RV-A59 (1)	8	Rhinovirus A89 (RV-A89)
Rhinovirus B	RV-B (1), RV-B86 (1)	2	Rhinovirus B14 (RV-B14)
Rhinovirus C	RV-C (4)	4	Rhinovirus C3 (RV-C3)

### Enterovirus VP1 antibody reactivity first declines until 6 months of age, and then it steadily climbs.

**FIG 1:**
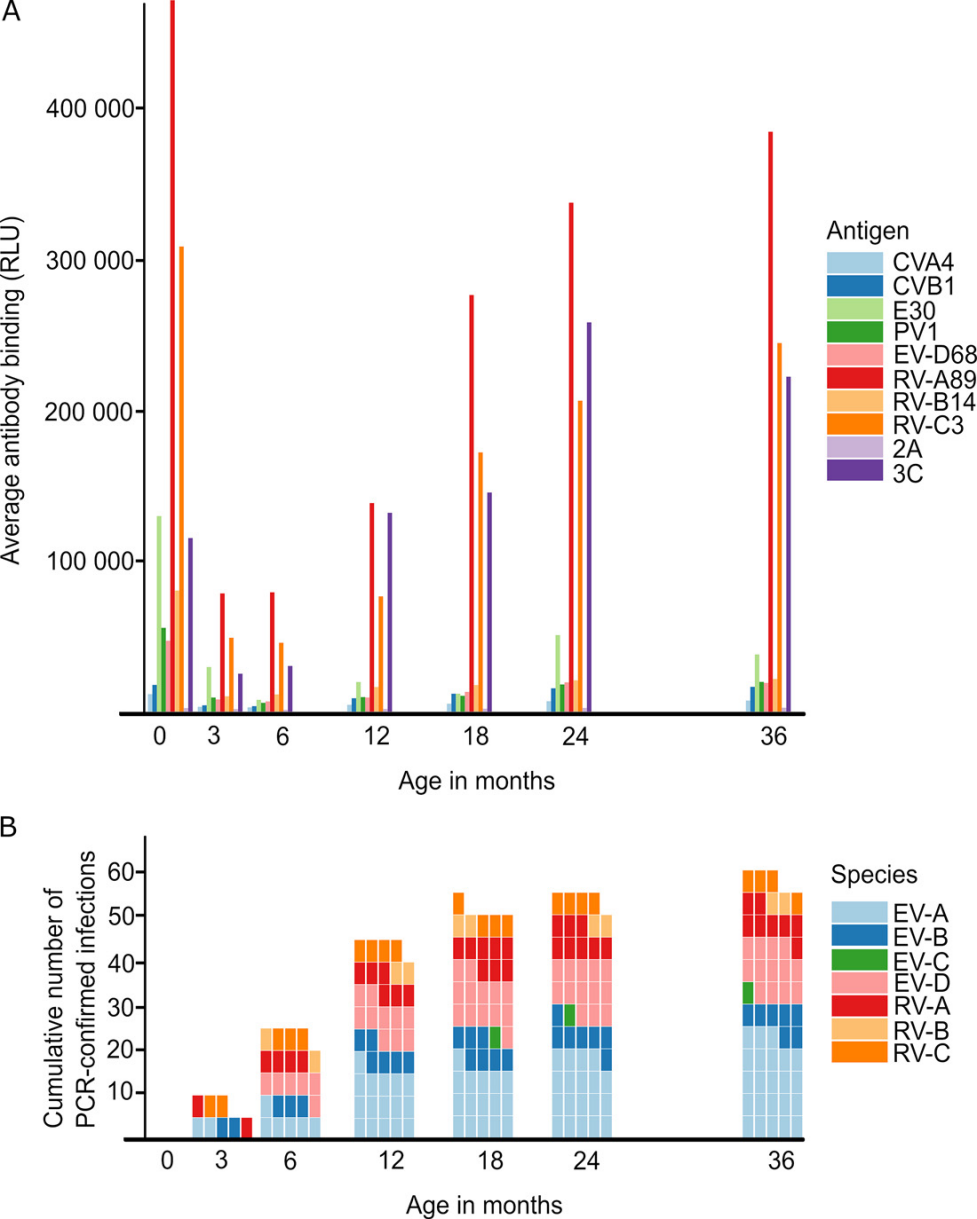
The average of VP1 and protease antibody signals for subjects from birth to 36 months of age (A) and the cumulative number of PCR confirmed enterovirus infections by species (B). See Fig. S1A for the data from panel A presented in a logarithmic scale for the easier comparison of low-level antibody responses.

To get an overview of the development of antibody levels in children over time, the signal averages for antibody responses to different Enterovirus antigens were plotted at each time point (VP1 proteins from each enterovirus species and proteases 2A and 3C from CVB3). An examination of the signal averages revealed the expected trend of antibody responses first diminishing from birth to 6 months of age, as the maternal antibodies wane. This was followed by a rise of the child’s own antibody level. The strongest responses were seen against the Rhinovirus A and C VP1 proteins and against the CVB3 3C protease (Fig. 1A). Interestingly, the 3C signal appears to decline at 36 months of age. The majority of PCR-confirmed infections occurred during the first year of life, whereas the proportion of infections by Enterovirus A species increased with age (Fig. 1B). The antibody levels against all of the enterovirus antigens rose as a function of the cumulative number of infections. However, this trend is the clearest for the 3C protease. Even if we include the 36-month sample, where the average 3C signal is starting to fall, we get a correlation coefficient (ρ) of 0.94 with a *P* value of 0.017 when doing a Spearman-rank correlation test (Fig. S1C).

### The antibody responses do not appear to be type-specific or species-specific.

**FIG 2:**
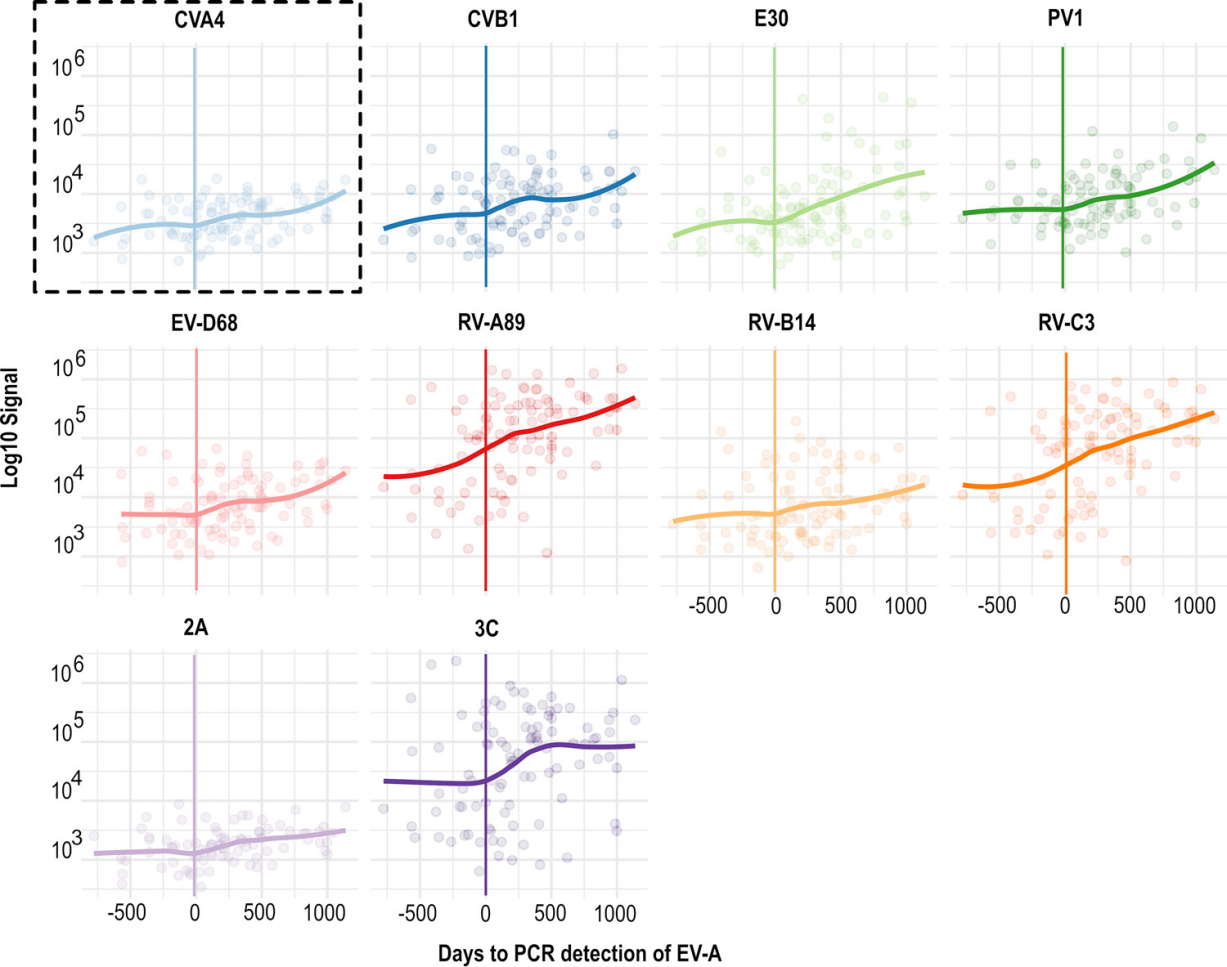
Trends in antibody responses against different enterovirus antigens around the time of a PCR confirmed Enterovirus A species infection (number of subjects = 23). Data points are filtered to include only measurements performed after the age of 3 months so as to reduce the influence of maternal antibodies. A vertical line marks the time when Enterovirus A was detected by PCR. See Fig. S2 for a similar graph plotted for the Enterovirus D-infected group (*n* = 13), which was the second largest infection group in the study.

We chose to study the children with Enterovirus A infection in detail, as they formed the largest infection group (*n* = 23). Using PCR results as a base, we temporally related the antibody data to the time of infection (Fig. 2) and used local regression to visualize the temporal trends. The antibody levels increased after an Enterovirus A infection against the matching CVA4 antigen and several other enterovirus types. The most marked increase was seen in antibodies against the CVB3 3C protein. Thus, it appears that the antibodies binding to the used enterovirus antigens cross-react widely between antigens that represent different enterovirus species. Figure S2 shows similar results for the Enterovirus D infection group.

### Antibody responses are more enterovirus type-specific when analyzed in individual children.

**FIG 3:**
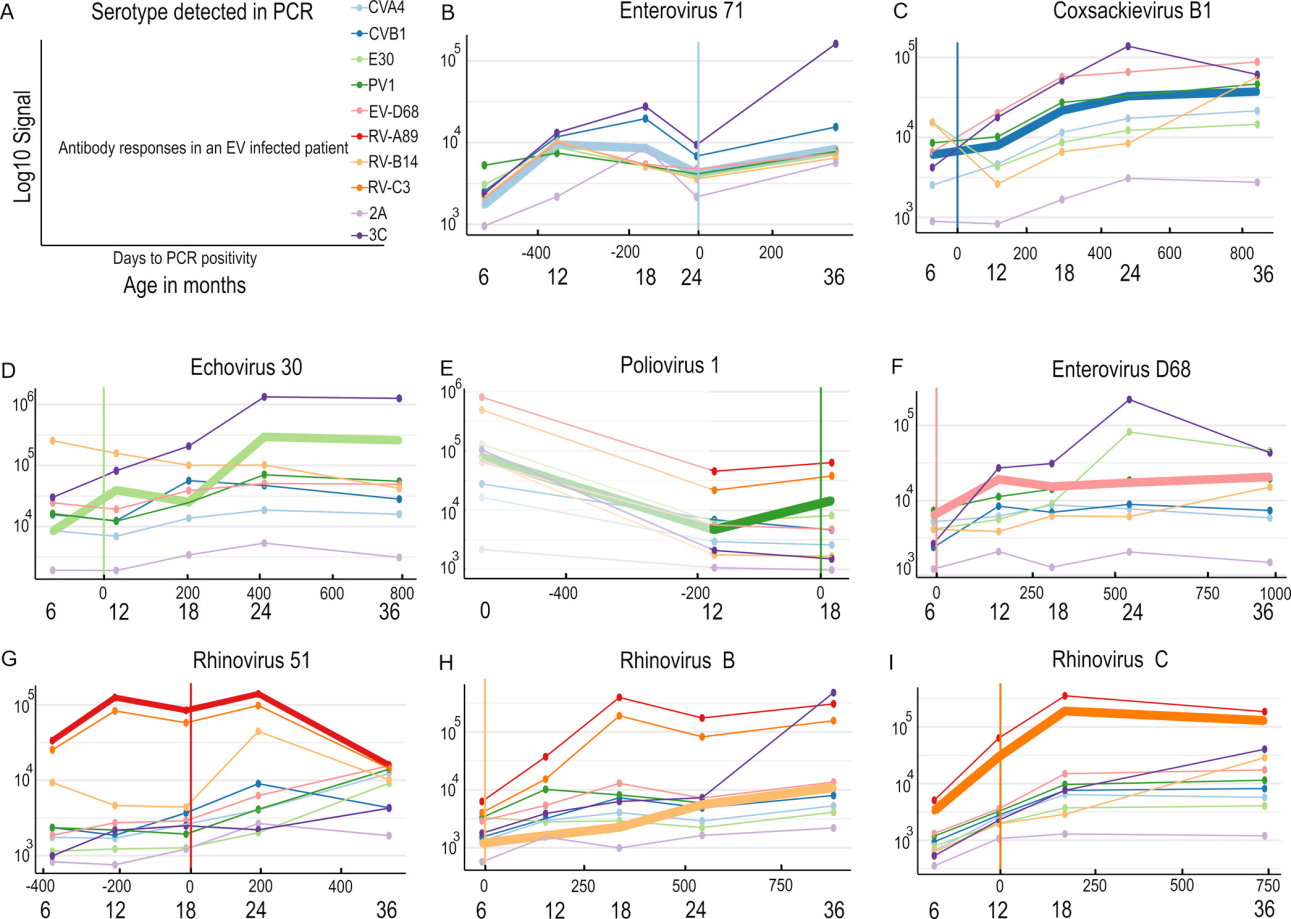
Antibody responses against enterovirus antigens for individual children from 6 months to 36 months of age. (A) The legend for reading the graph. (B–I) Individual subjects with a single, known, PCR-confirmed EV infection. A vertical line shows the time of the PCR-confirmed infection, and the thickest line represents the response to the VP1 that is most closely associated with the infecting enterovirus type. For subjects infected by those other than rhinoviruses, the RV-A89 and RV-C3 antigen responses are not shown. This is due to them overshadowing the other responses and making it hard to compare the weaker responses. For poliovirus 1, the cord blood sample is also shown in the graph with shading, as we only had one subject in our sample set that had PCR positivity to poliovirus 1 (PV1).

Going down to the level of individual children, we can see that the antibody levels increase after an infection and target a limited number of enterovirus antigens. However, the greatest change in antibody levels did not necessarily occur against the antigen that was most closely related to the infecting species or type (Fig. 3). In some cases, the antibody levels to certain VP1 proteins that were not matching to the enterovirus type causing the infection had already started to decrease around the time of infection, indicating more specificity. For instance, the CVB1 and E30 infected patients that are shown in Fig. 3C and D exhibited a rise against the PCR-confirmed enterovirus around the infection but a decline in RV-B14 and CVB1, respectively. Mirroring the results from the level of infection group, the 3C protease antibody levels seemed to increase in all Enterovirus infections. It is noteworthy to mention the close correlation between the RV-A89 and RV-C3 antibody responses, which were not plotted for all of the subjects due to them being universally high. Due to there being only one Enterovirus C positive subject (PV1) and only a few samples available from that subject, we plotted the cord blood sample into the graph, unlike for all of the other subjects (Fig. 3E).

### Cross-reactivity varies over time.

The cross-reactivity between different enterovirus antigens was evaluated in the same Enterovirus A infection group via a correlation analysis. We assumed that changes in correlation between the time points would indicate antigen specificity and that a nonspecific response would appear as similar correlation across all time points for antigen pairs. The pairwise comparison of antigen signals showed a high correlation between several of the antigen pairs, but this depended greatly on the time point. We observed two pairs of antigens with high correlation across all of the time points: RV-A89 and RV-C3 as well as PV1 and E30. Other than these, there is great variation in correlation between the antigen pairs at the different time points. For example, there is a negative correlation between the RV-C3 VP1 and protease 3C responses in the cord blood, but a positive correlation is observed as early as 3 months of age, and the positive correlation remains through the 3-year monitoring period. It also appears that the VP1s for Enterovirus A through Enterovirus D seem to form a group in which the members’ reactions are more correlated with each other than to those of the other antigens.

## DISCUSSION

### General trends seen in the data: the roles of maternal antibodies and early rhinovirus infections and the rise of Enterovirus A infections at later time points.

Challenges with the immunological assays that were conducted in the children in this age group involved the presence of maternal (IgG) antibodies (22) and the children’s own immune systems being in dynamic developing phases (23). The results from both the infection groups and the individual children suggest that before 6 months of age, until the maternal antibodies had disappeared, any possible rise in antibody levels of the children seemed to be masked by the declining maternal antibody levels, making serodiagnostics challenging. This is also apparent when comparing all of the signals (regardless of the infection group) in the cord-blood samples and in the six-month samples, where the difference is significant for all of the antigens (Fig. S5A). The antibody responses against rhinoviruses A and C were, on average, considerably higher than those for other enteroviruses, regardless of the nature of the confirmed infections. It also appears that in most cases, the subjects had a rise in multiple EV antibody levels around the time of PCR positivity, with the most conspicuous rise not always occurring for the antigen that is phylogenetically closest to the infecting agent. For instance, in Fig. 3C, the child with a PCR-confirmed CVB1 infection had a steeper rise in EV-D68 antibodies around the time of infection. Then again, it is entirely possible, that the child had an EV-D68 infection during the 6 months between the samplings.

### Basis for the cross-reactivity of antibodies binding to the VP1 proteins of different enterovirus types.

The relatively high correlation between VP1 responses was anticipated based on both literature and our previous work on testing antibody responses against a similar panel of enterovirus antigens with unpaired human serum samples (19). However, in our previous study (19), we noticed that cross-reactivity was more extensive in adults than in children, when comparing individual time points. In the current study, with samples spanning over a three-year follow-up period in young children, we studied whether the correlation between the PCR-confirmed enterovirus type and the antibody response against the corresponding antigen within that enterovirus group would be stronger. The overall correlation followed the same trend as the overall reactivity. The correlation between different VP1 antigens first dropped until the age of six months, as the maternal antibodies waned, and then they started to increase as the children experienced enterovirus infections.

The increasing cross-correlation of antibody responses against different VP1s could be attributed to the subjects having numerous enterovirus infections (7) and thus having antibodies against multiple EVs, or the original antigenic sin, in which existing responses are prioritized and enhanced over specific responses to new, related infections. The latter phenomenon has been soundly established for influenza viruses and is considered to be the reason for the need for seasonal vaccinations (24). The original antigenic sin has also been discussed by Niespodziana and colleagues in the context of Rhinoviruses in which the N-terminal section of VP1 is deemed to be the culprit (25). The first 14 to 20 amino acids in the Rhinovirus A and C VP1s are nearly identical, and the antibodies against this epitope can be found in the majority of the human population (18). Iwasaki et al. studied the potential of the Rhinovirus VP1 proteins for diagnostic purposes and found that the antibodies against Rhinovirus A and Rhinovirus C cross-reacted strongly (16). They also found that the antibodies in Rhinovirus A positive sera bound Rhinovirus C VP1 proteins more strongly than did the antibodies in Rhinovirus C positive sera. Once they removed the first 14 amino acids from the RV-C3 VP1, they identified more type-specific antibody responses. It is possible that this phenomenon explains the highly correlated Rhinovirus A and Rhinovirus C VP1 responses. The RV-B14 VP1 differs considerably in this region from the RV-A89 and RV-C3 VP1s (Fig. S3A), which may in turn explain how the RV-B14 results show a weaker correlation with the two other Rhinovirus VP1 responses.

In Enterovirus A through Enterovirus D (and RV-B14), the EV-group-common VP1 N-terminal epitope is highly conserved (Fig. S3), and it plays a significant role in the antibody cross-reactivity toward VP1 proteins. The entero-specific epitope of RV-B14 is more similar to those of Enterovirus A through Enterovirus D than to those of RV-A89 and RV-C3, which might explain some of the antibody response correlations that were observed between RV-B14 and the non-RV VP1s (Fig. S3A). As previously shown (19, 20), we observed that RV-B14 VP1 can be detected by our in-house monoclonal antibody (26), which targets the non-rhinovirus EV-specific PALTAVETGA-epitope in a Western blot (Fig. S3 and S4).

A recent study on maternal antibodies (22) reported that Rhinovirus A and Rhinovirus B as well as Enterovirus A through Enterovirus C are all among the 44 most commonly found virus antibodies in human sera. Of these sera, more than 50% react with the PALTAVETGA region and with enteroviral 2C helicase. More than 90% of the children at birth (or their mothers) were positive for peptides containing the first 50 residues of the VP1 proteins of several Rhinovirus A and Rhinovirus B species. These peptides coincide with the regions that have the rhino-specific and entero-specific epitopes, both of which are highly conserved.

The enterovirus capsid structure is thought to open on contact with a receptor or when the virus is immobilized on surfaces such as polystyrene, thereby revealing the VP1 N-terminal segment where the group common epitope is located. It has been shown that when using full virus capsids in sandwich ELISA, the results are more type-specific, compared to the direct coating of capsids on plastic (27, 28). This so called “antigenic shift” has also been reported in empty particles versus full ones when fractioning enterovirus particles via ultracentrifugation. It has been observed that the empty particle primarily binds IgG class antibodies, whereas IgM antibodies mostly bind to the full particle. Some enteroviruses have been reported to naturally produce large amounts of empty particles, and it has been speculated whether this is, in fact, enhancing the virus infection, as the antibody response targets non-infective particles (29). The immunodominance of the enterovirus group common epitope in adults is also interesting, as in our own studies in young children, the epitope (as a peptide) alone does not seem to elicit a strong reaction, whereas most adult sera recognize it. It is possible that the PALTAVETGA epitope itself is not highly immunogenic in young children, compared to the epitope that is common for the rhinovirus group, as reflected by the raw signal values in the current study. However, repeated exposure will amplify reactivity to the PALTAVETGA epitope. The current study is not able to state for certain whether the rhinovirus epitope is more immunogenic or whether the stronger reactivity is simply a result of more frequent rhinovirus infections at this age.

**FIG 4:**
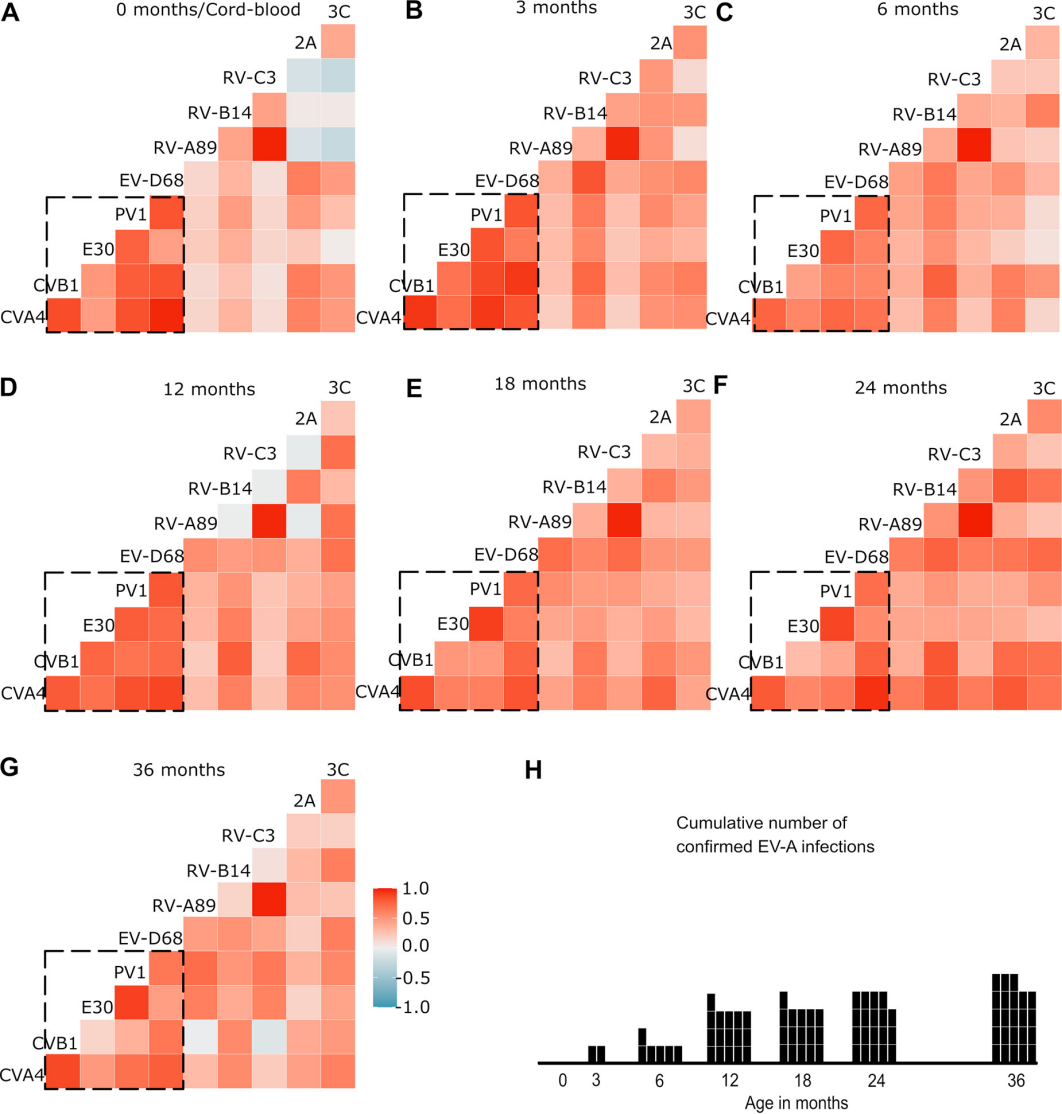
The Spearman correlations of antigen responses at different time points for Enterovirus A infected subjects. (A–G) The pairwise correlations vary across time, but some pairs (RV-C3 and RV-A89 as well as PV1 and E30) seem to correlate strongly, regardless of time points, indicating high cross-reactivity. (H) Cumulative number of PCR-confirmed Enterovirus A infections.

In the context of enteroviruses, poliovirus vaccination is one potential confounding factor. In Finland, poliovirus vaccines are part of the national vaccination program. The IPV vaccine is given at 3, 5, and 12 months of age. The one child in this study with a PCR-confirmed polio infection most likely got it from an oral polio vaccine (OPV). From the Enterovirus A infection group data in Fig. 4, we can see that PV1 and E30 reactivity remain highly correlated, even when the correlation between antigen pairs in the Enterovirus A through Enterovirus D block seems to even out with time. Interestingly enough, although the children had been vaccinated against polio, the PV1 responses were not as high as we expected. We have seen good responses against whole poliovirus capsids in earlier studies utilizing indirect ELISA. Comparing the whole virus reactivity to just the VP1 responses could shed some light on the matter. Be that as it may, the response toward the PV1 VP1 antigen gets stronger with age, as do most of the VP1 responses (Fig. S1A). One explanation could be that we see an effect that is similar to that which was described by Niespodziana et al. in 2012 for rhinoviruses, but in all enteroviruses, with the responses becoming more focused on VP1 over time (25).

### Antibody responses to nonstructural enterovirus proteins.

The responses toward the nonstructural proteins 2A and, especially, 3C seem to be elevated around the time of PCR-confirmed EV infection and could thus represent a widely reacting probe for EV infections. This finding is also in line with the results of our previous work on strong 3C responses in acute infections (19, 20). In our previous work, we noticed that when comparing antibody reactions to EV antigens, 3C reactivity was especially pronounced in children under the age of two years, compared to adults (19). Similarly, in our previous studies, we found the protease antibodies to be better markers for acute infection than VP1 antibodies in adults, and we observed that in CVB-infected mice, the protease antibody response was shorter-lived than the VP1 response (20). Unexpectedly, in the current study, we saw that the 3C response does not seem to decrease before 36th month. This could indicate that there are several enterovirus infections occurring during the first three years of life that continuously boost antibody responses to the 3C protein. This is supported by the fact that it is estimated that children have approximately four times as many rhinovirus infections annually, compared to adults (7). Alternatively, it might indicate that antibody responses toward the proteases are not as good indicators of ongoing or acute infections in children as in adults but are rather a good general probe for the previous enterovirus infection load. After all, the proteases are more conserved than the VP1s among enteroviruses (Fig. S3B and C). Nevertheless, the proteases appear to be interesting targets for immunological studies.

### Limitations of the study.

The sampling rate in this study was not optimal for studying the specificity of the responses. The kinetics of antibody responses are problematic, as it takes about 2 weeks from an initial infection for an antibody response to rise and go through affinity maturation and class change to mount a full-scale, high-affinity IgG antibody response. On a subsequent infection of the same virus, or of another related virus that is recognized by existing antibodies, the response takes less time. The half-life of an antibody response varies greatly for different viruses, but, for example, in COVID-19 vaccinated people, the level of neutralizing antibodies is halved every 108 days (30). In contrast, a recent study by Qiu et al. in 2021 mapped the kinetics of neutralizing antibodies in EV-A71 infected patients and found that neutralizing antibody levels halved in 26 months from their peak value at 14 days postinfection (11). In this study, we used an antibody that binds to the H+L part of the human antibody, which, in theory, should pick up all of the antibody classes. We have also previously seen that the neutralizing antibody response does not correlate well with detection antibodies in ELISA, which underlines the challenges associated with the serodiagnostics of viruses.

Due to the frequency of enterovirus infections in young children, it is impossible to know how many and which species of enterovirus infections the subjects may have had between samplings. For example, it is highly likely that the subject in Fig. 3F had an Enterovirus B infection near the 24-month sampling time point, but we do not have PCR confirmation for that event. While this cohort is good for seeing general trends, it is not well-suited for studying serodiagnostic accuracy: After leaving out cord-blood and three-month samples, we tried finding paired sera based on loose criteria by selecting serum samples at a maximum of 300 days prior to and 180 days after a PCR-confirmed Enterovirus A infection to test the significance of the change in the signal, but we were left with 5 cases (Fig. S5B). We have been using pooled sera from a small number of six-month-old children as a control due to the low reactivity to most enteroviruses at that age (Fig. 1A; Fig. S1B). The rhinovirus antigen reactivity remains markedly higher in the pool, compared to the Enterovirus A through Enterovirus D reactivities in the pool. Establishing a baseline reactivity in order to apply strict cutoffs with which to diagnose infections from single time point sera has proven difficult due to the great variance in reactivities between individual children. So far, we are limited to studying the fold changes in reactivities to specific antigens between sera that were collected at different time points. As a result, this tool is not yet suitable for use in routine diagnostics but can be useful in a research setting. To further study the specificity of the panel, we would need to have a more frequent serum sample series around known infections. Such studies have been performed in adult volunteers when it comes to the relatively harmless rhinoviruses, but we have no access to such sera. Ethically, it would be highly questionable to conduct such studies with pediatric subjects. Alternatively, the specificity could be studied in animal models, but this would be only in part, as not all human enteroviruses are capable of infecting other mammals. Rhinovirus infections (Rhinovirus C, in particular) have been challenging in mice models, and only recently have such models been developed (31). However, using vaccination, such a study against structural proteins in animal models could be partly covered, as several experimental enterovirus vaccines are available (32–36).

### Suggestions for future studies.

The average correlation between different VP1 protein responses seemed to decrease between time points of 24 and 36 months (Fig. 4), indicating that the VP1 panel might give more species-specific results after early childhood. Therefore, it would be interesting to study how the overall correlation between VP1 antigens and the antigen responses continue to develop during later childhood, as in our previous studies, we found that the protease responses were a good indication of an acute or ongoing enterovirus infection in adults (19, 20). In contrast, we saw in this study that the 3C protease response does not seem to stabilize before the 36-month time point after starting to rise due to an infection. Interestingly, the 2A response steadily climbed up after the six-month time point, even with a lower overall signal level, which might also render it as a valuable marker of enteroviral infections. It might also be worthwhile to study the antibody responses against other nonstructural proteins to find out whether they would be even more precise markers of acute enterovirus infections or the overall infection load. For instance, 2C antibodies have been found to be common in cord blood samples (22). Furthermore, it would be interesting to see if removing known cross-reactive VP1 N-terminal epitopes could make the assay more type-specific or species-specific. However, there is a risk here, as this might reduce the sensitivity of such an assay, especially in adults, in whom the majority of the response seems to be against these epitopes, at least for rhinoviruses (25). The antigens that we have used in this study (whose plasmids have been deposited to Addgene) are relatively easy to produce (or could be obtained from us for a nominal fee), and, as the MSD UPLEX platform is commercially available, it would be simple for other laboratories to set up and use this assay in their own studies. The cost of running this experimental assay for a single sample is roughly 6.5 euros (omitting labor costs), with the majority of the price coming from the MSD UPLEX kit. We will continue to add to and improve this panel of antigens in our future studies.

## MATERIALS AND METHODS

### Human samples.

347 follow-up serum samples from 58 children were obtained to test the specificity of the antigen panel. Each of the children chosen for this study met our criteria for having at least one enterovirus RT-PCR-positive stool or nasal swab sample for a single enterovirus species that coincided within the first three years of his or her life. The type was defined by sequencing the VP1 coding region of the virus (37). At least one sample before and after an enterovirus PCR-positive sample was available for antibody analyses. These serum samples were obtained from the birth cohort arm of the DiabImmune study and were collected in Espoo, Finland; Petrozavodsk, Russia; and Tartu, Estonia during the years 2008 to 2013 to study the pathogenesis of type 1 diabetes, celiac disease, and allergic diseases, to which EVs have been linked. The prospective serum samples in the cohort from 74 children in each country were collected when the children were 0, 3, 6, 12, 18, 24, and 36 months old. Details concerning the numbers of PCR confirmed infections and serotypes are summarized in Table 1. The children were originally chosen on the basis of carrying HLA-conferred susceptibility to T1D and/or celiac disease. All of the participants of the cohort study had the informed consent of their legal guardians. The study was approved by the Ethics Committee of the Helsinki and Uusimaa Hospital District, Helsinki, Finland (228/13/03/03/2008).

### Antigen production.

VP1 antigens were produced as in (19). Briefly, sequences coding for recombinant VP1 proteins were cloned into a pGEX-2T vector (resulting in an N-terminal fusion of GST). The plasmids encoding various VP1 proteins have been deposited to Addgene (https://www.addgene.org/Vesa_Hytonen/). E. coli (Star DE3) cells were transformed via heat shock and plated on LB-Amp plates. Seed cultures were prepared from single colonies and grown overnight at 37°C. The cultures were upscaled to two liters and grown to an optical density (600 nm) of 0.6 in LB supplemented with ampicillin and 0.5% glucose, after which protein production was induced with 100 nM isopropyl β-D-1-thiogalactopyranoside (IPTG), and the culturing was continued overnight at 25°C. Bacteria were pelleted via centrifugation, resuspended into PBS, and lysed via sonication in the presence of lysozyme. The lysate was clarified, and the soluble fraction was bound to glutathione agarose (Pierce glutathione agarose, Thermo Scientific) overnight at 4°C. The resin was washed with PBS, and the protein was eluted with 40 mM reduced glutathione in PBS. For the second affinity purification step, the elute was bound to Ni-NTA resin (HisPur Ni-NTA Resin, Thermo Scientific) for 2 h and washed with 50 mM imidazole in PBS, after which the protein was eluted with 500 mM imidazole in PBS. After ascertaining the purity of the proteins via SDS-PAGE and Western-blotting, the antigens were dialyzed into PBS to get rid of the imidazole and were biotinylated with a 20× molar excess of the EZ-link NHS-PEG12-biotinylation reagent (Thermo Scientific, Rockford, IL, USA, catalog number 35389). The excess biotinylation reagent was dialyzed away, and the biotinylation of antigens was confirmed using a derivative of the WB technique, in which neutralized chimeric avidin (38) was used for detecting biotinylated proteins on membranes and avidin antibodies were used for detection (38).

### Antigen quality control.

Antigen concentrations were measured using a BCA assay (Pierce) and UV-VIS-spectroscopy (NanoDrop One). The antigen purity, identity, and biotinylation were analyzed via the total protein staining of SDS-PAGE gels and detection via Western blotting using broadly recognizing anti-enterovirus antibodies (26), and neutralized chimeric avidin was used to detect the biotinylated proteins (exemplified in Fig. S4). Some of the purified proteins had multiple bands in SDS-PAGE. These samples were further analyzed via gel filtration, mass spectrometry, and mass photometry to confirm that the bands were either dimers or degradation products of the VP1-GST fusion proteins.

### Custom 10-plex MSD UPLEX assay and its optimization.

MesoScale Diagnostics (MSD) assays utilize electrochemiluminescence for signal production with a camera in their plate readers for detection and quantization from their proprietary well plates. MSD offers different multiplexing options, one of which (UPLEX) allows for the addition of linkers that guide any biotinylated target molecules to specific spots on the bottoms of the wells in their plates. In our custom assay, in-house antigens were first biotinylated and equipped with a unique UPLEX linker. After the linking reactions and subsequent coating of the spots in the wells, the assay was performed similar to a standard indirect ELISA, with the serum samples acting as the primary antibodies and the sulfo-tagged anti-human (H+L) antibody acting as the secondary antibody. The plates were read using a MSD Quickplex SQ-120 plate reader, and the data were exported to R for analysis.

The optimization for the 10-plex assay was performed as before (19). Briefly, the MSD UPLEX development kit was used to set up and run the assay. The extent of the biotinylation of the antigens was analyzed semiquantitatively via densitometry from the proteins that were blotted on the membranes and detected with neutralized chimeric avidin, using monobiotinylated BSA as a reference. The concentrations of antigens in the linking reaction was adjusted, based on the number of biotins on them. Following the instructions from MSD, we adjusted the concentration of the biotinylated antigen to a level that should ensure that most of the linkers (applied at a concentration of approximately 33 nM) had the target antigen bound prior to coating. Therefore, antigens with four biotins were linked at a 16 nM concentration, antigens with two conjugated biotins were linked at a 33 nM concentration, and monobiotinylated antigens were applied at a 66 nM concentration.

Pooled sera products, such as Nanogam and Hizentra, as well as pooled sera from six-month-old infants and several known positive samples were run using the antigen panel to test for a suitable dilution having the signal levels of the positive samples be between 1,000 and 1,000,000 for all of the antigens, according to the instructions from the MesoScale Diagnostics assay specialists. In the samples from the children, this dilution was between 1:1,000 and 1:10,000.

### Data processing and figures.

The statistical analysis and figure generation were carried out in R (39), using the following packages: ggplot2 (40), dplyr (41), reshape2 (42), ggpubr (43), GGally (44), RColorBrewer (45), and waffle (46). The final touches to the figures were made with Inkscape version 1.2. Pseudonymized raw signal data along with a script for a ShinyApp to visualize the data similarly to Figure 3 is available at the first authors github repository: https://github.com/NiilaJouppila/Enterovirus_antibodies_early_childhood.
