# Supplementary material for: Assessment of Enterovirus Antibodies during Early Childhood Using a Multiplex Immunoassay
Source: Microbiol Spectr. 2023 May 25;11(3):e05352-22. doi: 10.1128/spectrum.05352-22 (PMC10269870; doi:10.1128/spectrum.05352-22)
Supplement: Supplemental file 1 — Supplemental material. Download spectrum.05352-22-s0001.docx, DOCX file, 0.96 MB [file spectrum.05352-22-s0001.docx]

**Supplementary data**

**Assessment of Enterovirus antibodies during early childhood using multiplex immunoassay**

Jouppila N.V.V^1^., Lehtonen J^1^., Seppälä E^1^., Puustinen L^1^., Oikarinen S^1^., Laitinen O.H^1^, Knip M^2,3^, Hyöty H^1^., Hytönen V.P^1,4^.

^1^Faculty of Medicine and Health Technology, Tampere University, Tampere, Finland

^2^Research Program for Clinical and Molecular Metabolism, Faculty of Medicine, University of Helsinki, Helsinki, Finland

^3^Department of Pediatrics, Tampere University Hospital, Tampere, Finland

^4^Fimlab Laboratories, Tampere, Finland


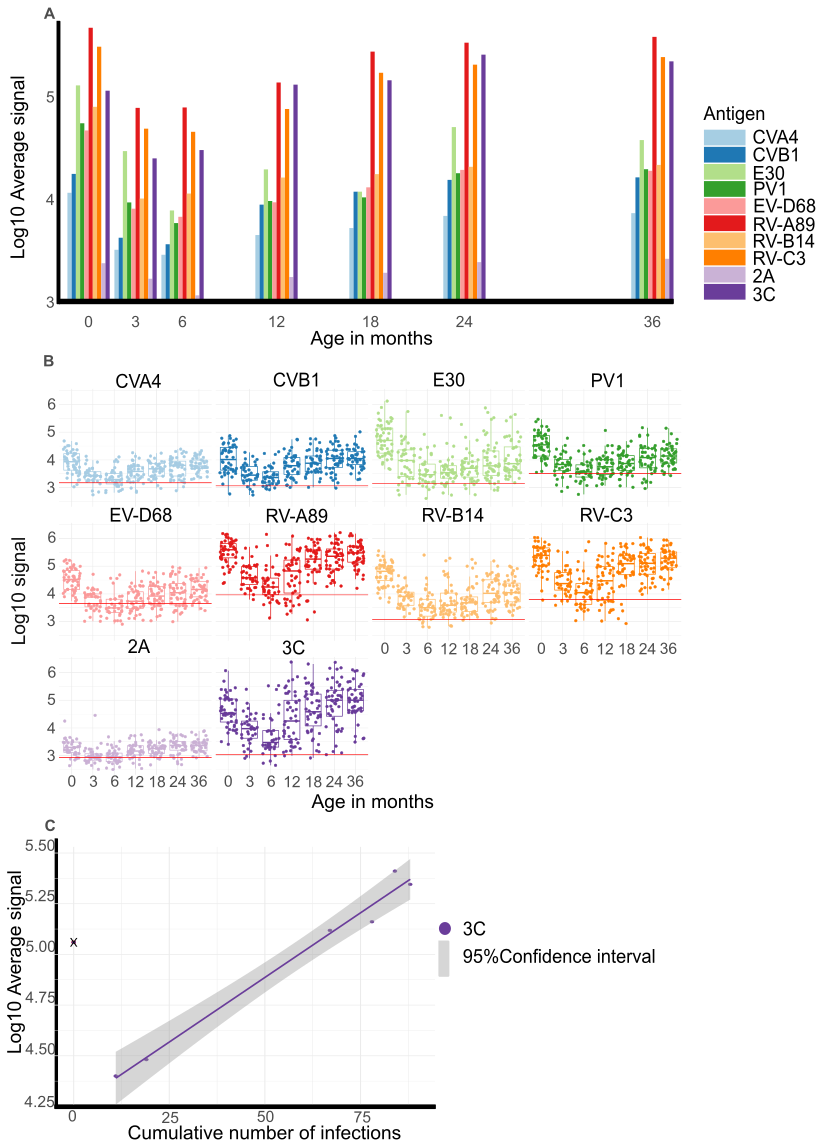


**Figure S1.** Average of VP1 and protease antibody signals for children in this study from birth to 36 months of age on a logarithmic scale for easier comparison of antigens with weaker signals (A). (B) Boxplots with data points representing the same data with signal level from our negative control (pooled 6-month-old sera) in red. (C) the linear model of 3C signal as a function of cumulative infections. Spearman correlation test gives rho=0.94 and *p*=0.017 when excluding the cord-blood sample (0 infections, but a lot of maternal antibodies).


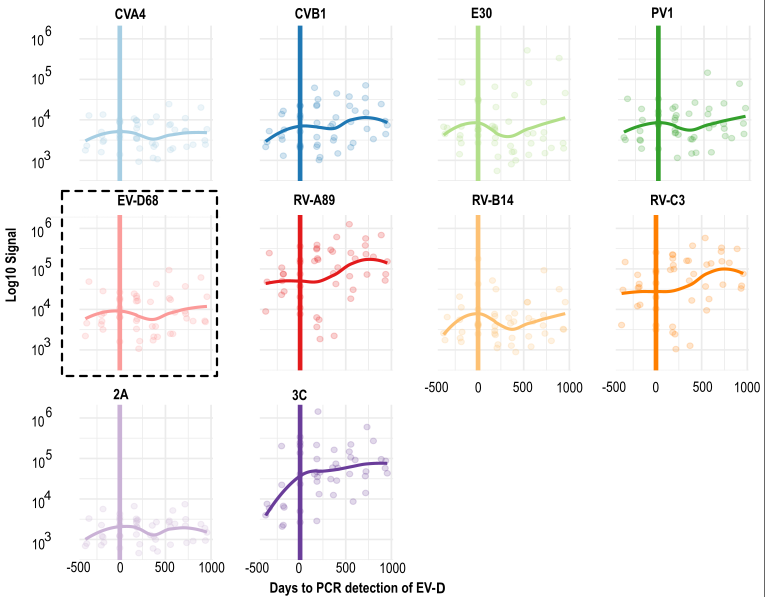


**Figure S2.** Trends in antibody responses against different enterovirus antigens around the time of a PCR confirmed EV-D species infection (number of subjects=13). Data points filtered to include signals for subjects after the age of 6 months to reduce the influence of maternal antibodies.


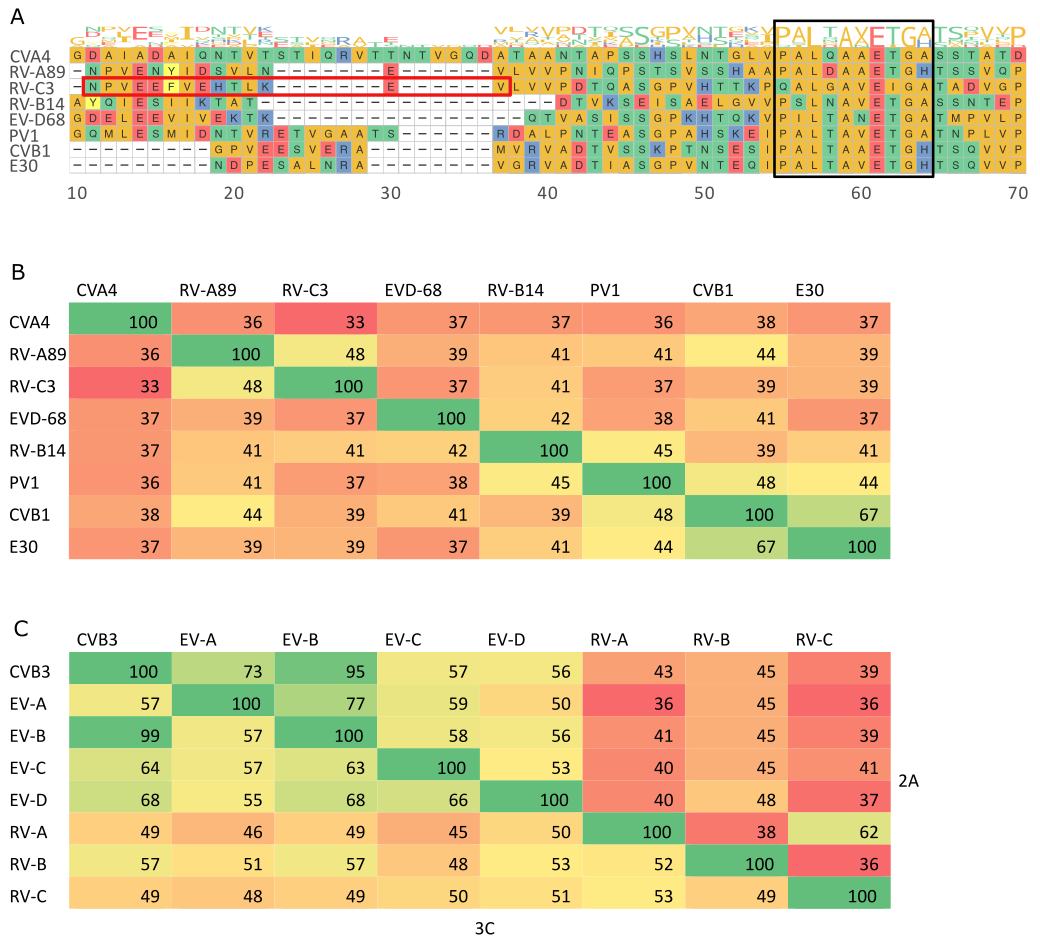


**Figure S3.** Alignment of the VP1 N-terminal sequences with the cross-reactive epitopes boxed (A). In the red box is the part Iwasaki et. al 2013 removed from RV-C3 VP1 to gain more type-specific responses (16) and in the black box is the enterovirus group reactive epitope. Percent Identity matrix for B) VP1 sequences used in the study (excluding GST and His-tags), as well C) for 2A and 3C proteases (upper and lower diagonals, respectively). Proteases used in this study are from CVB3 (2).

**Antigen quality**

GST-VP1 proteins were successfully produced, purified and biotinylated. VP1 proteins when run on gel had major bands at 60 kDa and 30 kDa. The lanes and bands in the blots are blurry possibly due to differing amounts of biotins on individual proteins. The theoretical sizes of the full recombinant proteins are ~60kDa depending on the construct. The VP1 proteins run on gel had bands both at ~60 kDa and ~30 kDa, the latter of which coincides with both the sizes of VP1 proteins and the GST-partner. Since we did not have antibodies capable of binding to all the VP1 proteins, we needed to confirm the identities of these bands by additional tests. Samples of VP1 proteins were run on size exclusion chromatography, which showed a single peak at around 60kDa for all the constructs. To confirm that both bands observed in electrophoretic analysis contained the protein of interest we used mass-spectrometry on PV1 VP1, which seemed to be the most unstable of the constructs. We confirmed that both the major bands contained parts of GST and the VP1 sequence. We also studied the PV1 VP1 protein using mass-photometry, which showed size distribution of the protein in solution to be 90% ~60kDA and 10% ~120kDa (GST tends to form dimers). After these tests we were satisfied on the quality of the antigens.


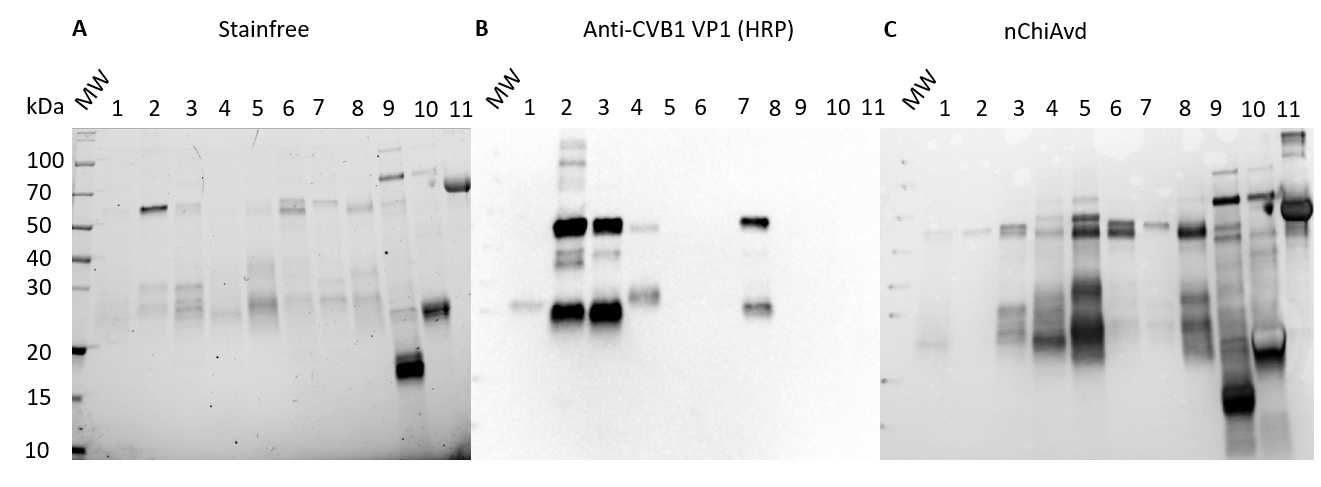


**Figure S4**. A) Total protein staining of antigens and BSA control. B) Rat anti-CVB1 VP1 (3A6, PALTAVETGA-epitope) staining. C) Detection of biotinylated proteins transferred on membrane using neutral chimeric avidin. 1.CVA4 2. CVB1 3. E30 4.PV1 5.EV-D68 6.RV-A9 7.RV-B14 8.RV-C3 9. 2A 10. 3C 11. monobiotinylated BSA.

We have shown in our previous studies, that some of the antigens are not stained properly using the stain-free stain, but stain normally with PAGEblue on gel, or Ponceau on membrane after the transfer (3). This may explain the observed poor detection of EV-D68 60 kDa band (Panel A, sample 5), for example.


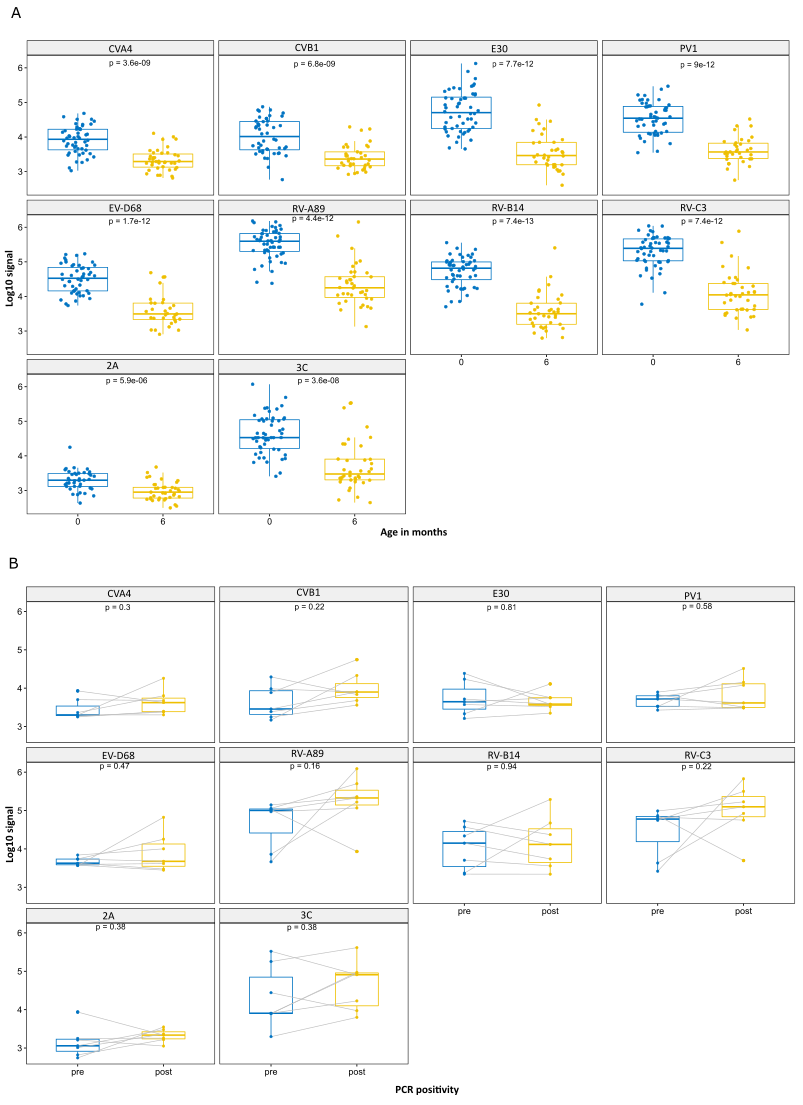


**Figure S5. Statistical assessment of antibody responses.** Antibody responses towards enterovirus antigens drop significantly from cord-blood samples until 6 months of age (A). Panel B) results for comparing samples taken before and after EV-A PCR-positivity in paired sera. Selection criteria: last serum sample prior to (max 300 days) and after (max 180 days) EV-A positive PCR sample and excluding the cord-blood and 3-month samples.

**Supplementary references**

1. Iwasaki J, Smith WA, Stone SR, Thomas WR, Hales BJ. 2013. Species-Specific and Cross-Reactive IgG1 Antibody Binding to Viral Capsid Protein 1 (VP1) Antigens of Human Rhinovirus Species A, B and C. PLoS One 8.

2. Laitinen OH, Svedin E, Kapell S, Nurminen A, Hytonen VP, Flodstrom-Tullberg M. 2016. Enteroviral proteases: structure, host interactions and pathogenicity. Rev Med Virol 26:251–267.

3. Saarinen NV V, Lehtonen J, Veijola R, Lempainen J, Knip M, Hyoty H, Laitinen OH, Hytonen VP. 2020. Multiplexed High-Throughput Serological Assay for Human Enteroviruses (vol 8, 963, 2020). Microorganisms 8:1426.
